# Supplementary material for: Design considerations for technology-assisted fall-resisting skills training trials in older adults: A pilot and feasibility study
Source: PLoS One. 2026 Mar 24;21(3):e0345798. doi: 10.1371/journal.pone.0345798 (PMC13012456; doi:10.1371/journal.pone.0345798)
Supplement: Attachment — Submitted filename: Response to Reviewers.docx [file pone.0345798.s003.docx]

Dear editors and reviewers, thank you for your time and attention taken on our manuscript and for your helpful comments. Below you will find our responses to each comment, first those from the editor and then reviewers 1 and 2. We hope these address your concerns and look forward to your feedback.

Editorial Comments:

Journal Requirements:

When submitting your revision, we need you to address these additional requirements.

1. Please ensure that your manuscript meets PLOS ONE's style requirements, including those for file naming. The PLOS ONE style templates can be found at <https://journals.plos.org/plosone/s/file?id=wjVg/PLOSOne_formatting_sample_main_body.pdf> and <https://journals.plos.org/plosone/s/file?id=ba62/PLOSOne_formatting_sample_title_authors_affiliations.pdf>

Authors’ Response: We have now adjusted the contents and files to adhere to the style requirements.

2. Thank you for stating the following financial disclosure:
This work was supported by the special research fund 2022 call for doctoral grants in the framework of BOF UHasselt – Maastricht University cooperation [BOF22DOCUM13].

Please state what role the funders took in the study. If the funders had no role, please state: "The funders had no role in study design, data collection and analysis, decision to publish, or preparation of the manuscript."
If this statement is not correct you must amend it as needed.
Please include this amended Role of Funder statement in your cover letter; we will change the online submission form on your behalf.

Authors’ Response: We have amended this as requested in the cover letter and manuscript.

3. Please include captions for your Supporting Information files at the end of your manuscript, and update any in-text citations to match accordingly. Please see our Supporting Information guidelines for more information: http://journals.plos.org/plosone/s/supporting-information.

Authors’ Response: We have added this as requested.

4. If the reviewer comments include a recommendation to cite specific previously published works, please review and evaluate these publications to determine whether they are relevant and should be cited. There is no requirement to cite these works unless the editor has indicated otherwise.

Authors’ Response: Understood, but not applicable in this case.

Additional Editor Comments :

The two reviewers have raised several important points. Please review all comments carefully and provide detailed responses.

Authors’ Response: Thank you. We have responded to all comments in a point by point manner below.


Reviewers' comments:

**Reviewer #1**: Thank you very much for this well written manuscript which gives a thorough background information on current topics in falls prevention as well as very helpful insights for the design of future training trials.
I have some minor concerns for you to consider:

**Authors’ Response:** Thank you for your thorough and helpful review of our work!

**Reviewer #1**: A small general remark: to my knowledge sentences like „… particularly in healthy older adults who do not necessarily yet feel, recognise or admit the need to participate in fall prevention interventions“ require a comma before the „or“ (bananas, peaches, or apples). Not being a native speaker myself, I have no strong feelings about this but maybe check. This would apply to several sentences in the text.

**Authors’ Response:** Thank you for this attention to detail. The Oxford, or serial comma, is typically considered optional. It is more common in American English and less common in British English, where it is typically only used to reduce ambiguity. Since the native speaker co-author is a British English speaker, we did not use it by default but we have reviewed the text to check for cases where its use would help improve clarity.

**Reviewer #1**: Introduction:

As community-dwelling older people are a very heterogenous group it might be helpful if you describe more precisely whether you are focusing exclusively on healthy, independently living older people without frailty, cognitive impairment, or a history of falls (primary prevention). This appears to apply to the sample of your pilot study. However, it would be helpful to know which of the citations you used in the introduction refer to this group. Additionally, it would be interesting if you focus solely on this group for your planned RCT.

**Authors’ Response:** The reviewer is correct that we focus exclusively on healthy, community-dwelling older adults, since, as implied in the introduction, our trial will take a primary prevention approach, though a recent history of falls is not an exclusion if they are otherwise healthy. We have now added a few additional details in the Participants section in the methods and included a link to the trial registry which lists the full inclusion criteria for the trial.

**Reviewer #1**: p.4. second paragraph: „For reactive balance during stance perturbations, the Stepping Threshold Test 27,28 is an example of a progressive, threshold based test that can analyse robustness of standing stability to perturbations of increasing intensity but an equivalent for GR that could objectively determine when a person deviates from “steady state gait” due to overt stability loss does not yet exist, though a dynamic version of the Stepping Threshold Test using subjective judgement of stability loss has been reported 29.“
This is a very long and complex sentence describing the important distinction between static reactive balance and dynamic reactive balance. Assessments of dynamic reactive balance control is difficult and so far, we do not have satisfying solutions. Please make at least two sentences of this important aspect.

**Authors’ Response:** We have now split this sentence into two separate sentences and also added a brief sentence explaining this further, as follows: “In contrast to stance, the body configuration is frequently unstable during the normal gait cycle, making precise assessment of when stability is lost more complex during walking.”

**Reviewer #1**: Methods
Experimental set-up: The sentence “For two tasks (boat, city ride), these six markers were covered and two shoulder markers were added to control the application.“ Is not very clear for the reader at that point. Although it is self-explicatory with further reading it might be helpful to rephrase, e.g: „For the two weight-shifting tasks (visual boat and car ride), these six markers were covered and two shoulder markers were added to control the application.

**Authors’ Response:** We have rephrased as suggested by the reviewer.

**Reviewer #1**: Experimental procedure:
I think it very interesting and important that you used the participant’s stability -normalized walking speed rather than preferred speed (maybe explain why). However, please make the sentence clearer to understand: “These trials were recorded and during a rest period following the familiarisation, participants’ stability-normalised walking speed was calculated (see calculation procedure in “Data processing” section below) and used for all following walking tasks to ensure that all participants walked at approximately similar stability during all tasks.“ Make it clearer, that you calculated each participant’s individual stability-normalized walking speed. Possibly you could already give a hint how this was achieved at this point, e.g.: „These trials were recorded and a rest period following the familiarisation was used to calculate the individual stability-normalised walking speed (resulting in a MoS of 0.05m, see calculation procedure in “Data processing” section below) for each participant which was used for all following walking tasks to ensure that all participants walked at approximately similar stability during all tasks.

**Authors’ Response:** We have slightly adjusted this section as suggested to improve the clarity.


**Reviewer #1**: p.7: obstacle avoidance test: please clarify whether and how it was measured whether the participant successfully avoided the obstacle. What happened if the participant stepped on or touched the virtual obstacle? Was there a perturbation or any form of feed-back?

**Authors’ Response:**  The details on calculation of the obstacle avoidance was included in the data processing section, as follows: “Obstacle hits were determined using the Center of Pressure (CoP) data recorded by the force plates. CoP signals were filtered using a second order low-pass Butterworth filter with a 15 Hz cut-off frequency. Obstacle position data was interpolated to the sampling frequency of the CoP. Stance phases were identified using a vertical ground reaction force threshold of 50N. The CoP trajectory was compared with the location of the moving obstacles projected onto the treadmill belt to identify whether the obstacle was hit or not. A hit was registered if the CoP position fell within an obstacle. For slow approach speeds (e.g. 0.5 times the treadmill speed), only the CoP positions immediately following foot placement were analysed.” There was no form of feedback when participants hit the obstacles since, at least applying perturbations, would then train an additional fall-resisting skill.

**Reviewer #1**: Is this obstacle avoidance task not planned for the RCT? Or will it be adapted according to the data of the pilot trial? (I ask, as you explicitly point out, that the stepping stone task will be used in the RCT.)

**Authors’ Response:** Perhaps it was missed but we did include the following statement in the Experimental Procedure section: “The first PGA task was an obstacle avoidance task, to inform the design of the PGA assessment and training tasks in our future trial.” So similar to the other tasks, this was a pilot version of the assessment that was intended for use in the trial. It was indeed our plan to adapt it if the pilot study results would indicate this to be necessary.

**Reviewer #1**: p.7: subjective feeling of stability: Please explain why you used a 7-point scale (a 10 point numerical rating scale is used for many applications e.g. pain). Or is it a Lickert scale?

**Authors’ Response:** It was indeed a 7-point Likert scale, since this seemed to be able to give us enough detail in responses while still being easy for participants to distinguish between the three points either side of 0. We also wanted to allow for nuance on both sides of 0. However, we did not have strong scientific reasons to choose this over another method, it simply seemed to suit the needs of this pilot study.

**Reviewer #1**: table 1: it would be helpful to the reader to repeat the aims in an abridged form in the legend of the table: e.g: 1) are non-task-specific balance tasks potential control tasks; 2) obstacle parameters affect difficulty; 3) determining a stability loss threshold for GR assessment; 4) effect of perturbation task unpredictability on anxiety; and 5) feasibility of various fall-resisting skill tasks

**Authors’ Response:** Thank you for this suggestion. We have added these to the table.

**Reviewer #1**: p. 11, second paragraph: This sentence lacks a noun, I believe: “The
descriptively and visually compared the outcomes of the three assessment methods (MoS, participant perception, and researcher evaluation), as well as the agreement between the gait researchers.“

**Authors’ Response:** We have corrected this.


**Reviewer #1**: Results

p.12: “11 older adults were recruited in this study.“ Avoid starting a sentence with a digit. “with a mean of 2 falls.“ Better: with a mean of two falls. Avoid digits for numbers <10.

**Authors’ Response:** This has been corrected

**Reviewer #1**: Missing data: please outline how you are going to avoid missing data in the planned trial. How many complete data sets did you gather? If I understand correctly 1 dropped out in the obstacle test due to knee pain, 1 only first half of task due to technical issues, 1 GR test stopped, 1 poor data quality, 3 no video data. I cannot tell if these are all different participants. If this were the case that would mean 7 incomplete data sets out of 11? Please describe your troubleshooting.

**Authors’ Response:** Thank you for this important comment. In the supplement, we provided a table with an overview of the missing data and the *n* of complete data for each task. In some cases, the errors due to technical reasons have already been addressed, either by altering the scripts of the applications to improve stability and by addressing a memory issue that affected the video recordings. So we do not expect these to be an issue in the trial. While we appreciate the suggestion to describe how we will mitigate these issues in the trial, we prefer to deal with this topic in detail in our trial protocol paper, which we are currently writing, since we do not feel that the pilot paper is the best place to present these details. However, we have added a brief sentence in the limitations, as follows: “In part, these errors could later be corrected by altering the scripts in the applications and by addressing a memory issue related to saving the video files to ensure that these would not occur again in the trial.”

**Reviewer #1**: p.16: consider rephrasing this sentence “However, as the percentage
increase in belt speed during the perturbation increased, some discrepancies occurred.“ (perhaps: However, as the percentage increase in belt speed during the perturbation was raised, some discrepancies occurred)

**Authors’ Response:** We have rephrased this as suggested.

**Reviewer #1**: „These did not occur at the highest intensities, where both researchers consistently identified visible gait adjustments and deviations from normal gait, but rather at moderate intensities, where it was sometimes unclear
whether a recovery step had occurred.“ Is this not what one would expect? You do not discuss this in the discussion.

**Authors’ Response:** This is not exactly what we had expected, though we must be honest here and say that we did not make any clear *a priori* predictions. We expected that there would be most inconsistency between researcher observations for the lowest intensities around where the first reactive recovery step occurs, since we would have expected these to be the more subtle movements. However, this is not what we observed since the agreement between observers for the first reactive recovery step was high. It was rather the intensities after this in the middle range that seemed to show less consistent agreement, as can be seen in figure 3D around the 35 to 60% points. In order to make this more clear in our writing, we have further adjusted this passage, which now reads: “The researchers mostly agreed upon the first occurrence of a recovery step. However, as the percentage increase in belt speed during the perturbation was raised beyond the occurrence of the first recovery step, some discrepancies occurred. These did not occur at the highest intensities, where both researchers consistently identified visible gait adjustments and deviations from normal gait, but rather at moderate intensities, where it was sometimes unclear whether a recovery step had occurred.”


**Reviewer #1**: p.19, 5th line from the bottom: „something was happening but couldn’t place exactly what.“ Please replace couldn’t with could not.

**Authors’ Response:** This has been adjusted

**Reviewer #1**: p.19, 3rd line from the bottom and last line: “5 participants mentioned they felt a bit nervous or anxious after the first RGR task with AP perturbations.“ „While the other 6 participants did not report any nervousness, anxiety or stress during this task.“ Please do not use digits for numbers < 10. Please check the whole text for this, also 3rd and 5th line of page 20.

**Authors’ Response:** This has been adjusted

**Reviewer #1**: Figure 5: in the legend correct please: RGR: reactive gait recovery (no capital letters)

**Authors’ Response:** This has been adjusted


**Reviewer #1**: Discussion

Page 21, second paragraph: Consider rephrasing ”for our first aim, …”. Maybe this would be clearer: “The tests we conducted for our fist aim showed that the three non-task-specific balance tasks …”

**Authors’ Response:** Thank you for this suggestion. We have rephrased this as follows: “The results related to our first aim indicated that the three….”

**Reviewer #1**: Page 22, second paragraph: “… the percentage increase in belt speed during the perturbation increased.“ to avoid using the word increase twice maybe rephrase: „… the percentage increase in belt speed during the perturbation was raised.“

**Authors’ Response:** This has been adjusted as suggested.

**Reviewer #1**: Page 22, second paragraph: “Previous studies using the same setup and perturbation method have shown that deviations from unperturbed MoS do not necessarily occur only on the first post perturbation step 30,51 Therefore, future research could consider a multi-criterion threshold to improve the accuracy and reliability of the approach. For example, we could expand the number of steps included in the MoS determined threshold to two or three to capture all possible types of responses to the perturbation, as described above“.
To support this point: In a study comparing young adults with older adults with or without a history of falls Hackbarth et al. (Experimental Gerontology 2025) showed that whereas in young adults step width and length normalize after only 1 or 2 steps in the older adults groups step width stayed elevated beyond step 8 after perturbation.

**Authors’ Response:** Thank you for highlighting this paper. Since it concerns step width and not a direct measure of stability, we will not incorporate it here. However, it might be worth mentioning in our protocol paper where we present the updated method that will be used in our trial.


**Reviewer #1**: Page 22 , last paragraph: „Regarding the participants’ perceptions, these did not align well with either the MoS threshold or with the researchers’ observations. Additionally, there was not perfect alignment between the participants responses to the different questions. These findings align with previous research of Kapur, et al. 68 who similarly found that older adults often have difficulty accurately judging their own balance.“
Consider replacing the word „align“ by another expression in one of the sentences.

**Authors’ Response:** We have replaced the last instance with “agree”.

**Reviewer #1**: Page 22, last paragraph: „For future trials, the implication of these findings is that older adults may not give objectively correct answers when asked about their stability and therefore cannot be relied on as a method for evaluating or progressing the difficulty of assessment or training tasks“ – I am not sure I can agree with this statement. It implies that there is one correct estimation of balance control ant that the people themselves cannot assess it correctly. Maybe we should consider two separate constructs, one the objective competence in reactive dynamic balance (for which we do not yet have a good measure) and the other the subjectively rated feeling of being in control of the person, which would be a concept of self-efficacy. We also know the objective fall risk and self-rated falls efficacy (e.g. measured with the FES-I) do not necessarily align but are constructs that interact.

**Authors’ Response:** Thank you for these points. We agree that these are separate constructs but we also think that we are quite clear that we refer to and are concerned with actual stability, which, if we are concerned about evaluating and improving fall-resisting skills, is the outcome we need to accurately monitor. We therefore feel that our statement is accurate. However, to help clarify this, we have added the following to the end of that sentence: “…despite these responses providing useful information regarding their balance confidence or falls efficacy”.

**Reviewer #1**: Page 23, first paragraph: “One general consideration of this GR task is that with the repeating perturbations, there may be an inherent learning effect that benefits participants.“ As there are several PBT trials that show that rate of laboratory falls decreases within the training, consider phrasing this sentence stronger, e.g.: … an inherent training effect seems probable …” The implication of this is that the outcome of the test includes not only the absolute GR of the participant, but also their ability to learn and improve their responses within the test. This is unavoidable but should be kept in mind when interpreting the outcomes.“ – I totally agree.

**Authors’ Response:** Since the intensities of the perturbations used during much of the GR task are much lower than those used in PBT trials and do not cause stability loss, at least at the beginning, we do not feel that the learning effects will be as strong as seen in PBT. Therefore, we do not feel that this sentence needs to be phrased in a stronger manner.

**Reviewer #1**: Page 23, second paragraph: “For the fourth aim, our results suggested, in contrast to our hypothesis, that with increasing unpredictability, anxiety levels decreased.“- it would be easier for the reader if you made two sentences, e.g.: „For the fourth aim, we hypothesized that with increasing unpredictability, anxiety levels might increase. However, anxiety levels decreased as over the course of the training perturbations became more unpredictable.“

**Authors’ Response:** We have adapted this as suggested.

**Reviewer #1**: Page 23, limitations: please include the selected study population as a limitation. Your results do not necessarily transfer to frailer, fall prone populations.

**Authors’ Response:** Since our pilot study was explicitly aimed at informing a trial in healthy, community-dwelling older adults, we do not consider this a limitation, since it was not our aim to generalise our results to more frail populations in which primary prevention is no longer applicable. However, to emphasise this for readers, we have added the following statement in the limitations section: “Finally, readers should take care in applying or extrapolating these results to the assessment, training or trials in other populations, since the current pilot study and our own related trial focus explicitly on healthy, community-dwelling older adults.”

**Reviewer #2**: This manuscript reports a pilot study that examined the feasibility of, and several key design considerations for, technology-assisted fall-resisting skills training trials in older adults. Prior to publication, several points should be clarified. My comments are as follows.

**Authors’ Response:** Thank you for taking the time to review and comment on our manuscript!

**Reviewer #2**: 1. Gait Robustness (GR) and Reactive Gait Recovery (RGR) may influence each other. Was it possible in this study to evaluate these two constructs separately? Please clarify how GR and RGR were operationally distinguished in the study design and analyses.

**Authors’ Response:** To some extent, we partly agree with the first statement of the reviewer. Based on the definitions we provide in the paper, it is possible that GR may affect RGR, but only in an indirect manner. Specifically, a better GR means that a larger perturbation is needed to destabilise the person, meaning that, for a given perturbation, better GR can mean that RGR may be easier, though this is not necessarily guaranteed. For example, in some clinical populations, it is known that they walk with greater MoS (and therefore increased GR) presumably as a preventative measure since their RGR is negatively affected by their condition (see section 5 in “Notes on the margin of stability” for a full account of this phenomenon: https://www.sciencedirect.com/science/article/pii/S0021929024001222#s0045 ). So while GR may indirectly influence RGR, they are theoretically different, independent constructs. Further, RGR by our definition cannot affect GR, since the former only occurs once stability is lost and the latter is in play prior to stability loss. In our study, the thresholds at which GR was typically being determined was at a lower perturbation intensity than at which RGR was being assessed, since we chose a perturbation intensity for the RGR tasks that we knew from multiple previous studies would consistently cause stability loss in all participants, which further distinguishes the skills/tasks. Note though that GR is a relatively less well studied construct and we did already note in our introduction that it is not well understood in practice how distinct these skills are from each other; this is one of the primary research questions of our main trial.

**Reviewer #2**: 2. In the GR test, what instructions were given to participants regarding how to respond to the perturbations? In addition, what was the rationale for separating the GR task from the RGR task? Could reactive balance ability have been evaluated within the GR task itself? Please explain why the authors considered it necessary to evaluate GR and RGR using distinct tasks.

**Authors’ Response:** Participants were not instructed on how to respond to the perturbations. They were simply asked to try to continue walking as normally as possible. Regarding the second question, we had separate aims and hypotheses related to perturbation unpredictability and anxiety that needed to be addressed separately. In addition, we wanted to distinguish clearly between the different tasks for the participant to also get their experience of the different tasks to assess aim number five.

**Reviewer #2**: 3. In the GR test, the perturbation direction appears to mimic a trip. If so, the margin of stability (MoS) quantified here may reflect only balance loss in the forward direction. Is it necessary to also consider backward balance loss (e.g., slip-like perturbations), which would challenge stability in the opposite direction? Please justify the focus on the current perturbation direction and clarify the scope/limitations of MoS interpretation in this context.

**Authors’ Response:** Thank you for this question, which is a complex one. Firstly it is good to clarify that, in our main trial, we consider multiple outcomes and perturbation directions, but the assessment tasks and outcomes that are our primary outcomes are similar those presented here. Due to these being our primary outcomes in the trial, this pilot study focused on those. Secondly, in general, our assessment and training approach in the trial focuses on obstacle-induced falls as a primary target, which, as the reviewer correctly points out, typically occur primarily in the sagittal plane with stumbles/trips causing a forward loss of stability. We believe that these are among the more common, but importantly more preventable/trainable causes of falls. Our trial protocol goes into this in more detail. Since the current pilot study is not directly an intervention study itself, it did not seem appropriate to spend a lot of text on this specific issue, but hopefully this response clarifies this for the reviewer. Whether or not it is “necessary to consider backward balance loss”, as the reviewer asks, is a matter of perspective we think. In general, fall prevention approaches should of course consider these types of falls as well, being the second most common cause of falls, but slip-related falls are not the focus of our approach. Second to the obstacle-focused approach, it is also a practical issue in that slips are much harder to accurately simulate on a treadmill compared to stumbles and trips. For this, an overground setup with slippery surface elements would be more ecologically valid.

**Reviewer #2**: 4. In Fig. 2, what does a negative MoS indicate? Does it represent balance loss in the forward direction, in the backward direction, or does it indicate loss of stability in the anteroposterior (AP) direction? Please provide a clear explanation of the sign convention and how it should be interpreted.

**Authors’ Response:** Figure 2 presents the SD-normalised MoS value (AP), since we evaluated the 3SD threshold as a method to evaluate GR. The dotted lines at +3 and -3 on the y-axis indicate this threshold. So this graph does not communicate any absolute value of balance loss but the MoS relative to the SD of the 10 steps prior to the perturbation.

**Reviewer #2**: 5. The manuscript states: “MoS values are normalised in SD based on MoS during 10 steps of normal walking before each perturbation.” Why were MoS values normalized by the standard deviation (SD)? Please provide the rationale with supporting references for this normalization approach.

**Authors’ Response:** The explanation for this approach was mentioned in the introduction: “In some previous studies, the number of recovery steps to return to baseline walking stability was evaluated as the number of steps to return to within 0.05m of the mean margin of stability of the 10 steps prior to the perturbation ^30,31^. The group later updated this to a margin of stability value three standard deviations or greater away from the mean value since an individualised method was preferable compared to an absolute threshold due to individual differences in the variability of steady state gait ^32^.” We have added this information now also to the methods section after the explanation of the MoS calculation for clarity, as follows: “For the GR assessment task, 3SDs of the participant’s 10 steps prior to each perturbation was calculated to determine the threshold. This individualised threshold was used to account for individual differences in MoS variability ^32^.”.

**Reviewer #2**: 6. Previous studies have suggested that balance control in the mediolateral (ML) direction is more challenging than in the anteroposterior (AP) direction, and that older adults have particular difficulty in controlling ML balance. In the present study, the evaluation appears to focus on AP stability only. Please discuss the implications of not assessing ML stability and consider adding brief discussion of ML-related outcomes/limitations in light of prior literature (e.g., the references below).
<https://doi.org/10.2522/ptj.20090125>
<https://doi.org/10.1016/j.apmr.2008.01.023>
<https://doi.org/10.1093/ageing/afl078>
<https://doi.org/10.1038/s41598-022-18382-7>

**Authors’ Response:** As mentioned in our response to comment 3 above, our main trial will additionally assess ML MoS and assess and train mediolateral tasks, but these are secondary outcomes. For this pilot study, we focused on our primary tasks and outcomes. As mentioned above, our primary focus in the trial is obstacle-induced falls – the most common cause of falls in healthy, community-dwelling older adults. While some studies show deficits in mediolateral balance control, the epidemiological evidence overwhelmingly suggests a need to train fall-resisting skills in the context of avoiding, resisting and recovering from obstacles. There is also video evidence (Yang, Y., et al. (2016). "Risk factors for hip impact during real-life falls captured on video in long-term care." Osteoporosis International 27(2): 537-547.) that landing on the hip after a fall (often presumed to be related to poor mediolateral balance) is equally likely to occur when the initial direction of the fall is forwards compared to sideways and an initially forward fall direction significantly increases the odds of landing sideways. Therefore, we do feel that our focus on forward stability loss as our primary direction of interest is justified.
